# Bibliometric trends and emerging frontiers in RNA interference research for mosquito control (2010–2025)

**DOI:** 10.3389/finsc.2026.1758530

**Published:** 2026-03-17

**Authors:** Nina Ghislaine Yensii, Fabrice Banadzem Kernyuy, Theophilus Nang Wakai, Titilope Modupe Dokunmu, Olubanke Olujoke Ogunlana

**Affiliations:** 1 Department of Biochemistry, College of Science and Technology, Covenant University, Ota, Ogun State, Nigeria; 2 Covenant Applied Informatics and Communication - Africa Centre of Excellence (CApIC-ACE), Covenant University, Ota, Ogun State, Nigeria

**Keywords:** bibliometric analysis, dsRNA, mosquito, RNA interference, vector control

## Abstract

**Background:**

Mosquito-borne diseases, such as malaria, dengue, and Zika, pose significant global health challenges, intensified by rising insecticide resistance and environmental concerns associated with conventional control methods. RNA interference (RNAi) offers a promising, eco-friendly, and species-specific approach for mosquito vector control by silencing critical genes. This study aims to assess the research landscape of RNAi in mosquitoes through a bibliometric analysis.

**Methods:**

Relevant publications from January 2010 to October 2025 were retrieved from the Web of Science and Scopus targeted RNAi-related keywords. Only peer-reviewed, English-language original research articles were included. Data were analyzed using VOSviewer for network visualization, Bibliometrix for bibliometric metrics, and Microsoft Excel for descriptive analysis.

**Results:**

The analysis included 480 articles, revealing a steady increase in publications, with a peak in 2020. The United States (45.4%) and China (22.1%) led research output, while African countries were notably underrepresented. Keyword co-occurrence analysis indicated a shift from foundational gene function studies to applied technologies, including nanoparticle and yeast-mediated RNAi delivery systems. High-impact journals like Proceedings of the National Academy of Sciences and Parasites & Vectors were prominent publication venues. Most studies targeted several genes, with the majority in Aedes species, then Anopheles species, and the least Culex species.

**Conclusion:**

RNAi research in mosquitoes has advanced significantly, evolving toward practical vector control solutions, yet challenges persist in delivery efficiency and field application. The lack of African representation highlights the need for equitable global collaboration. Strengthened international partnerships and increased regional investment are essential to realize RNAi’s potential for sustainable, effective mosquito control.

## 1 Introduction

Mosquitoes are among the deadliest animals on Earth, serving as major vectors for a range of debilitating and often fatal human diseases, including malaria, dengue, chikungunya, Zika, and yellow fever. These diseases impose a staggering global burden, with an estimated 700 million infections and 1 million deaths annually worldwide (1, 2). For instance, malaria alone caused approximately 249 million cases and over 608,000 deaths in 2024, predominantly affecting children in sub-Saharan Africa (3). Dengue fever has seen explosive growth, with over 4 million cases and more than 2,500 deaths reported from 101 countries in the first nine months of 2025 alone. Similarly, chikungunya outbreaks have resulted in 445,271 suspected and confirmed cases and 155 deaths globally between January and September 2025. In Europe, mosquito-borne diseases like West Nile virus are also on the rise, with 335 locally acquired cases and 19 deaths reported across eight countries as of August 2025 (4). These statistics reveal a hidden escalating threat, exacerbated by climate change, urbanization, and global travel, which contribute to the rise in mosquito proliferation and disease transmission.

Traditional vector control approaches, such as insecticide-treated nets, indoor residual spraying, larviciding (5) have historically reduced disease incidence by targeting mosquito populations at various life stages. However, these methods face serious setbacks due to widespread insecticide resistance, which has been documented in major malaria vectors across regions like Cameroon from 1990 to 2017 (6). Furthermore, chemical insecticides often lack species specificity, leading to unintended harm to non-target organisms and posing significant ecological risks, including biodiversity loss and environmental contamination (7). The World Health Organization (WHO) has highlighted the urgent need for innovative, sustainable alternatives to combat these limitations and achieve global health targets, such as reducing malaria mortality by 90% by 2030 (8). Other strategies that are being explored include the use of potential inhibitors (9, 10) and RNA interference (11).

RNA interference (RNAi) is a conserved post-transcriptional gene regulation mechanism found in eukaryotic organisms, first described by Fire and Mello in Caenorhabditis elegans (12). RNAi is initiated through the introduction of double-stranded RNA (dsRNA) or small interfering RNA (siRNA), which triggers the degradation of target messenger RNA (mRNA) and suppression of protein expression. In molecular entomology, it has been widely exploited as a powerful and environmentally friendly tool for functional gene analysis and pest control strategies (13). This conserved biological process has revolutionized functional genomics and holds immense promise for pest management due to its precision, minimizing off-target effects compared to broad-spectrum chemicals (14).

In mosquitoes, RNAi has been extensively applied to study and disrupt critical physiological processes, including gene function, reproduction, immunity, and vector competence—the ability of mosquitoes to transmit pathogens (14, 15). Recent advancements have transitioned RNAi from laboratory tools to practical vector control strategies. For example, oral RNAi delivery systems using yeast-based larvicides have shown efficacy in silencing genes essential for mosquito survival, such as those involved in larval development, with controlled-release formulations enabling sustained control of Aedes aegypti populations in large water containers (13, 16). Microalgal-based RNAi larvicides have also been developed to target mosquito larvae, providing an eco-friendly method to reduce populations of species like Anopheles and Aedes (17, 18). Additionally, nanoparticle-mediated RNAi has enhanced dsRNA delivery, improving gene knockdown efficiency for traits like insecticide resistance or pathogen transmission (19). In Europe, RNAi is being explored to combat invasive species like the Asian tiger mosquito (Aedes albopictus), with potential for field applications through innovative tools like RNA-based biopesticides (20). These developments highlight RNAi’s potential as a next-generation tool, aligning with WHO recommendations for novel interventions with unique modes of action.

Despite the growing body of RNAi research in arthropods, including general pest control applications (21) no comprehensive bibliometric reviews have focused specifically on mosquito studies. Bibliometric analysis is necessary for such studies because it provides a quantitative framework to evaluate the scientific landscape, track the evolution of research trends, identify influential contributors, and pinpoint gaps in knowledge that hinder practical applications, thereby guiding strategic research investments and policy decisions to address global health challenges like mosquito-borne diseases (21, 22). Such analyses are essential for mapping publication trends, identifying influential contributors, and uncovering thematic hotspots and gaps, which can inform future research priorities and policy decisions (21). Given the rapid evolution of RNAi technologies and the persistent global threat of mosquito-borne diseases, such an analysis is timely to assess progress and highlight underrepresented areas, such as field implementations in endemic regions.

This study aims to bridge that gap by conducting a bibliometric analysis of RNAi research in mosquitoes from 2010 to October 2025. Specifically, it examines publication trends, leading authors, institutions, and countries; performs citation and journal impact analyses; explores keyword co-occurrences to identify emerging hotspots; and evaluates the translation of RNAi technologies into practical vector control strategies. By doing so, this work seeks to guide researchers, funders, and policymakers toward advancing sustainable mosquito management solutions.

## 2 Methods

### 2.1 Data source

A comprehensive bibliometric analysis was conducted using a multi-database search approach. Scopus and Web of Science are now the most extensively used literature databases in almost all fields (23). In this study, the Web of Science Core Collection (WoSCC) database and the document search in the Scopus database (https://www.scopus.com) were used to generate a significant volume of papers in RNA interference (RNAi) studies on mosquitoes. WoSCC and Scopus were selected because of their broad coverage of high-impact, peer-reviewed scientific publications and robust citation indexing, making them ideal for bibliometric evaluations (24).

### 2.2 Search strategy

The search strategy combined variations of the terms *“RNAi,” “RNA interference,”* and *“gene silencing”* with the term *“mosquito.”* Specifically, a topic search (TS)—which retrieves records from titles, abstracts, author keywords, and Keywords Plus fields—was applied using the following Boolean query in WOSCC:

(“RNAi AND mosquito”) OR (“silencing AND mosquito”) OR (“dsRNA AND mosquito”) OR (“siRNA AND mosquito”) OR (“shRNA AND mosquito”) OR (“gene knockdown AND mosquito”).

For the Scopus database, the same search keywords were used, but limited to mosquitoes only.

The time frame spanned January 2010 to October 2025. Only peer-reviewed, English-language original research articles were included, while review papers, conference abstracts, and studies restricted to mosquito cell lines were excluded.

### 2.3 Inclusion and exclusion criteria

Studies were included if they met the following conditions:

- Original research articles published in peer-reviewed journals between January 2010 and October 2025.
- Written in English.
- Focused on RNA interference (RNAi) applications in mosquitoes, including studies on gene silencing, functional genomics, or vector control using dsRNA, siRNA, or shRNA.
- Conducted directly on mosquito vectors (e.g., *Aedes aegypti*, *Anopheles gambiae*).

Excluded studies include:

- Review articles, conference proceedings, or non-peer-reviewed publications.
- Articles published in languages other than English.
- Studies using mosquito cell lines instead of live mosquito vectors.
- Research on RNAi conducted in non-mosquito organisms.

All retrieved articles were manually screened by two independent co-authors to ensure compliance with these criteria. Any discrepancies were resolved through discussion and consensus to maintain data reliability.

### 2.4 Data extraction and analysis

The bibliographic data from selected publications were exported in BibTeX, CSV, and plain-text formats from WOSCC and in CSV format from the Scopus database for processing and analysis.

Data analysis employed a combination of bibliometric software tools:

- VOSviewer (v1.6.20) for mapping co-authorship networks, citation relationships, and keyword co-occurrence.
- Bibliometrix (via the Biblioshiny web interface) for descriptive and advanced bibliometric analyses, including country productivity, author performance, and journal impact.
- Microsoft Excel for data cleaning, descriptive statistics, and visualization of temporal publication trends.

**Figure 1:**
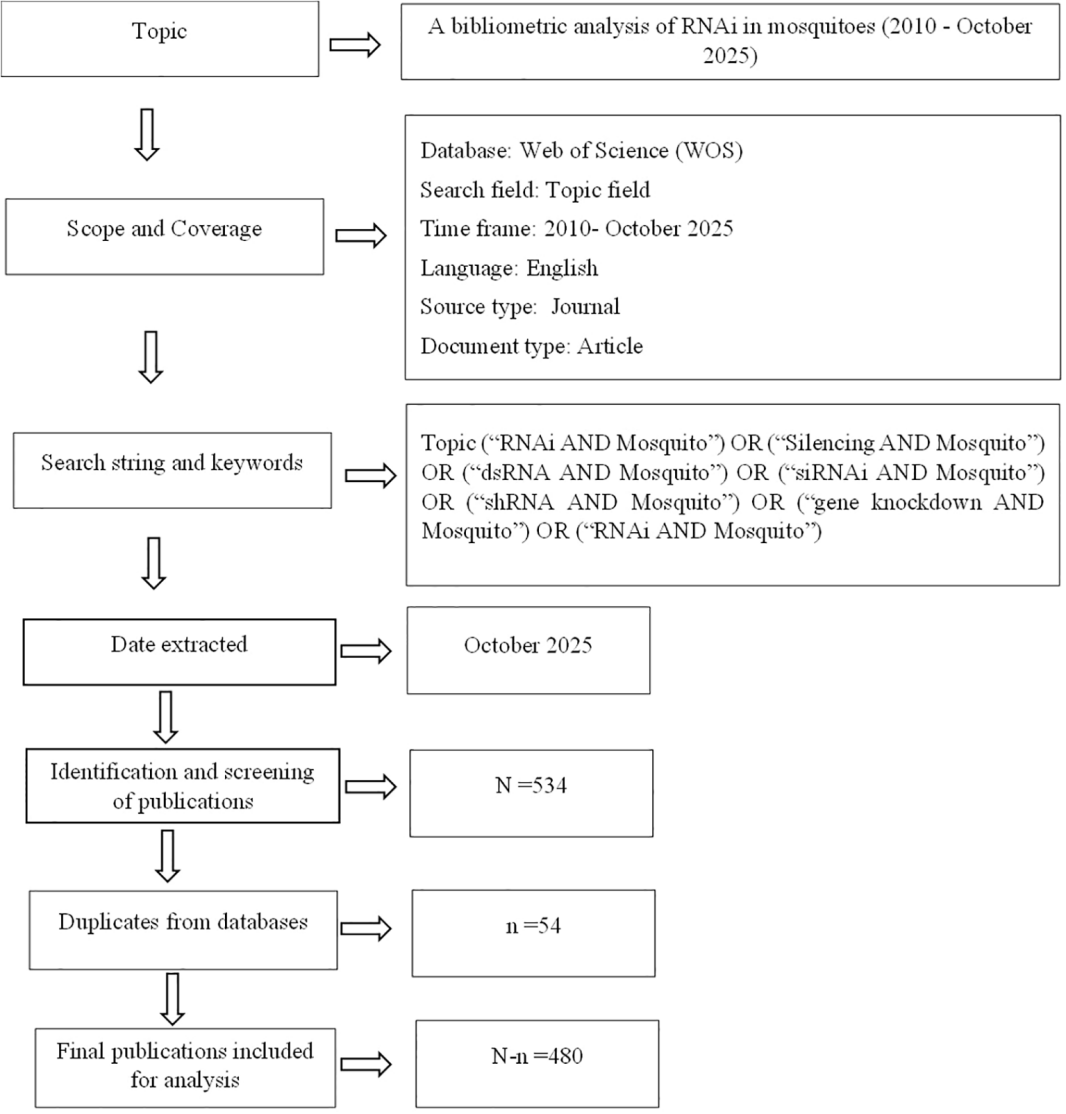
A flow chart of the bibliometric analysis.

Figures and tables summarize the major findings, including annual publication output, country and institutional productivity, journal performance, and thematic keyword evolution. **Figure 1** presents an overview of the search design and analytical workflow adopted in this study.

## 3 Results

### 3.1 Annual scientific publication

**Figure 2:**
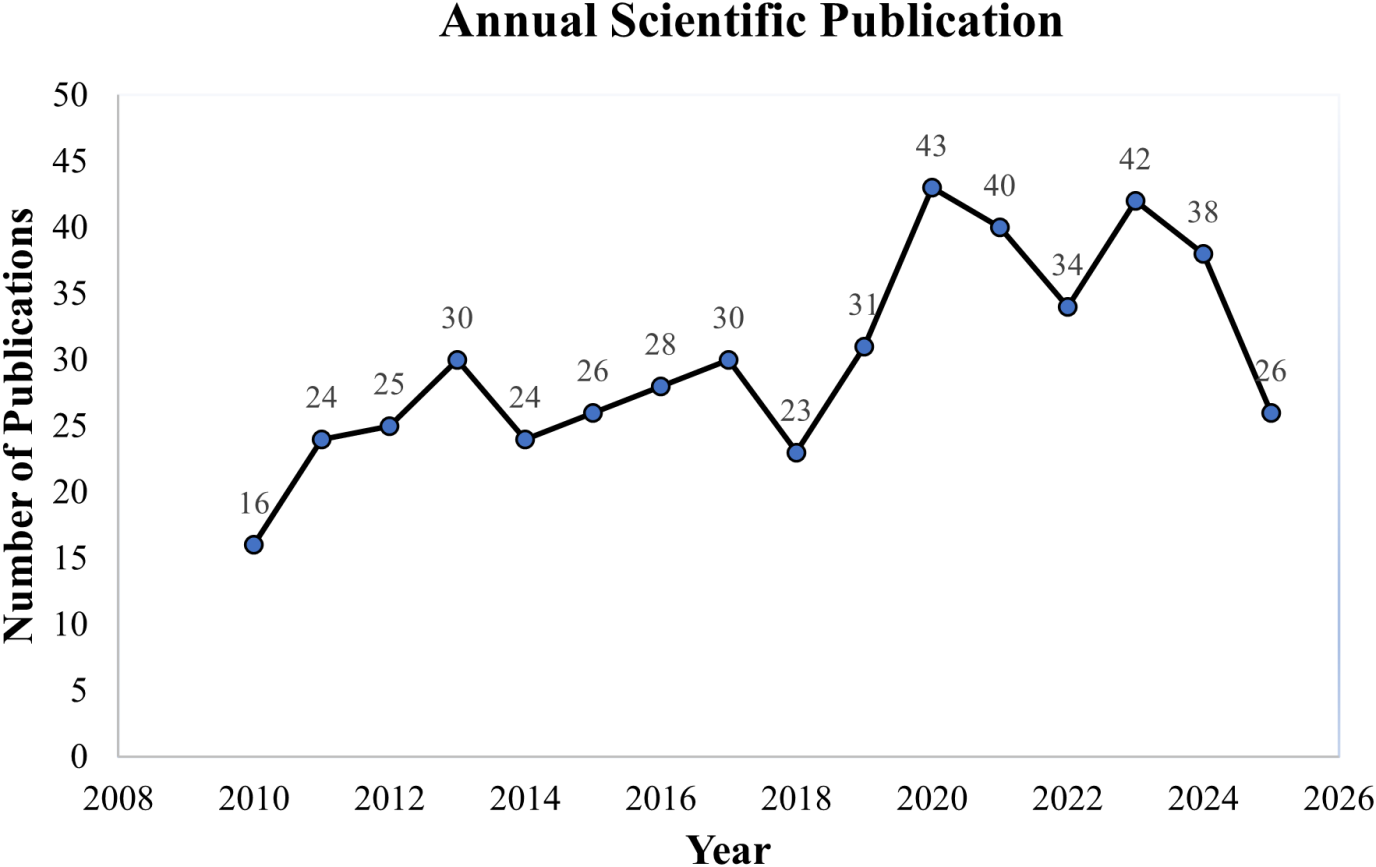
A line graph showing the number of publications per year.

From 2010 to October 2025, a total of 480 peer-reviewed articles on RNA interference (RNAi) in mosquitoes were identified through the Web of Science and Scopus databases. This reflects growing interest in this innovative vector control approach. Annual publication trends showed a consistent upward trajectory, increasing from 16 articles in 2010 to a peak of 43 in both 2020, before a decline to 26 in 2025, likely due to the data collection cutoff in October (**Figure 2**).

### 3.2 Analysis of publications based on countries

**Figure 3:**
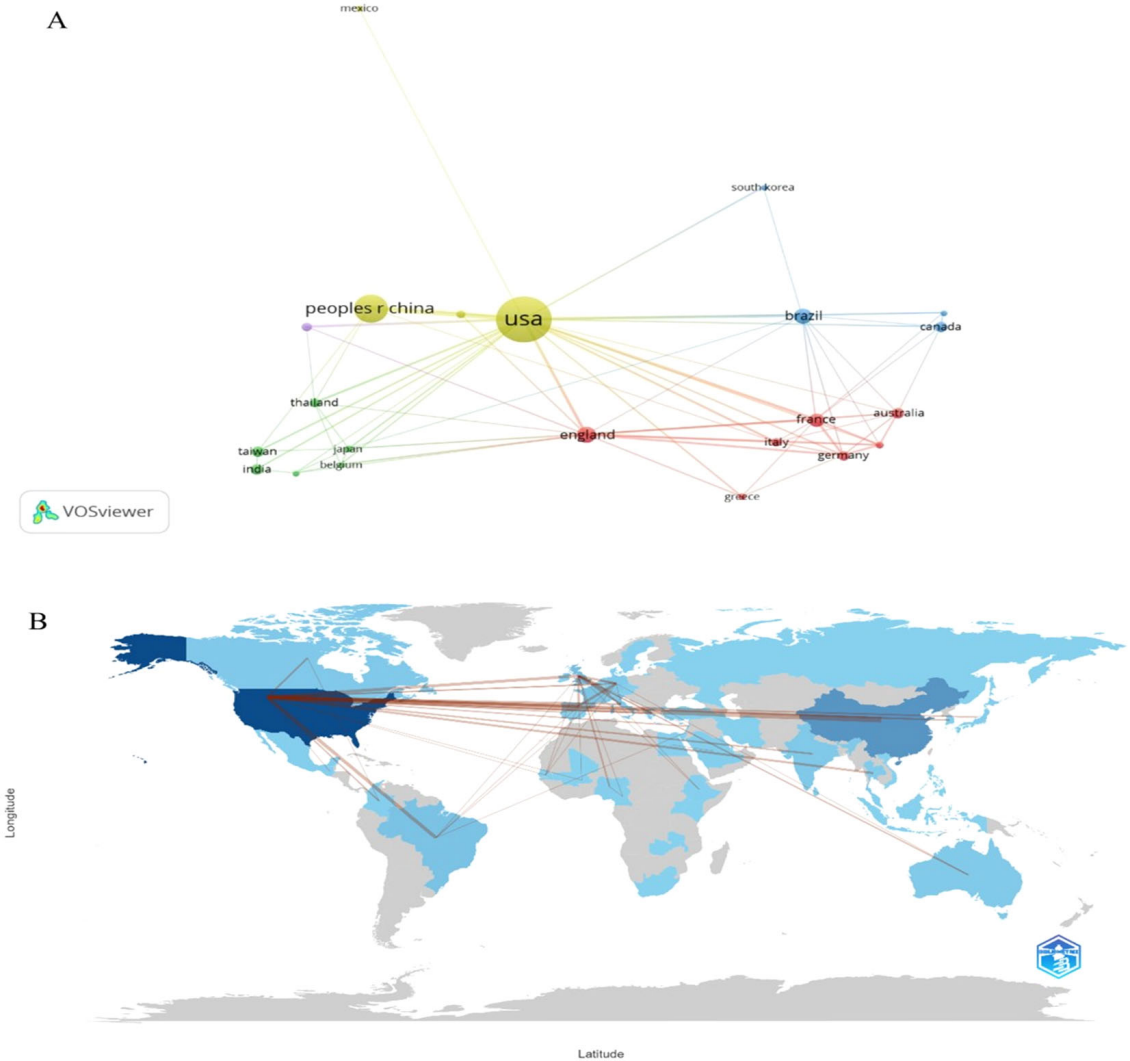
International collaboration network in RNA interference research on mosquitoes. **(A)** Network visualization **(B)** World collaboration map.

The global distribution of RNAi research in mosquitoes revealed significant regional disparities. The United States led with 45.4% of total publications (n=218), followed by China with 22.1% (n=106). Other notable contributors included the United Kingdom (n=19), Brazil (n=19), India (n=17), Canada (n=14), Australia (n=11), France (n=10), Lebanon (n=7), and Thailand (n=7). Notably, no African country ranked among the top ten, highlighting a critical gap given the high burden of mosquito-borne diseases in Africa (World Health Organization, 2024). Single-country publications (SCPs) dominated in the USA (72% of its output) and China (70%), while the UK and Brazil had higher proportions of multi-country publications (MCPs), indicating stronger international collaboration (**Table 1**). A VOSviewer network visualization of country collaborations (minimum five papers per country) showed the USA as a central hub, with strong links to China (link strength=39), the UK, and France, whereas China’s collaborations were primarily with the USA (**Figure 3**).

**Table 1:** Top ten productive countries in RNAi mosquito research.

Rank	Country	Articles	Articles %	SCP	SCP%	MCP	MCP %
1^st^	USA	218	45.4	169	77.9	48	22.1
2^nd^	China	106	22.1	77	77.8	22	22.2
3^rd^	United Kingdom	19	4.0	5	26.3	14	73.7
4^th^	Brazil	19	4.0	7	41.2	10	58.8
5^th^	India	17	3.5	10	62.5	6	37.5
6^th^	Canada	14	2.9	8	57.1	6	42.9
7^th^	Australia	11	2.3	10	90.9	1	9.1
8^th^	France	10	2.1	5	50	5	50
9^th^	Lebanon	7	1.5	2	28.6	5	71.4
10^th^	Thailand	7	1.5	4	66.7	2	33.3

Key: USA, United States of America; SCP, single country publication; MCP, Multiple country publication.

### 3.3 Analysis of publications based on institutions

Institutionally, the University of Notre Dame was more frequent in over 98 publications, followed by the University of California, Riverside (n=45), and Indiana University School of Medicine (n=40), underscoring the USA’s dominance in mosquito RNAi research. China’s Nanjing Medical University and Hainan University ranked fifth (n=37 each), reflecting the country’s growing capacity in molecular entomology, followed by the Universidade Federal do Rio De Janeiro of Brazil. Other notable institutions included Institut Pasteur Paris (France, n=30), Kansas State University (USA, n=30), Johns Hopkins University (USA, n=28), and Vanderbilt University (USA, n=28). The top ten institutions contributing to RNAi research in mosquitoes are shown in **Table 2**.

**Table 2:** Top ten institutions and their publication counts from 2010 to October 2025.

Rank	Affiliation	Publication frequency	Country
1^st^	University of Notre Dame	98	USA
2^nd^	University of California Riverside	86	USA
3^rd^	Indiana University School of Medicine	50	USA
4^th^	Universidade Federal do Rio De Janeiro	38	Brazil
5^th^	Hainan University	37	China
5^th^	Nanjing Medical University	37	China
6^th^	Institute Pasteur Paris	30	France
6^th^	Kansas State University	30	USA
7^th^	Johns Hopkins University	28	USA
7^th^	Vanderbilt University	28	USA

### 3.4 Analysis of publications based on authors

**Figure 4:**
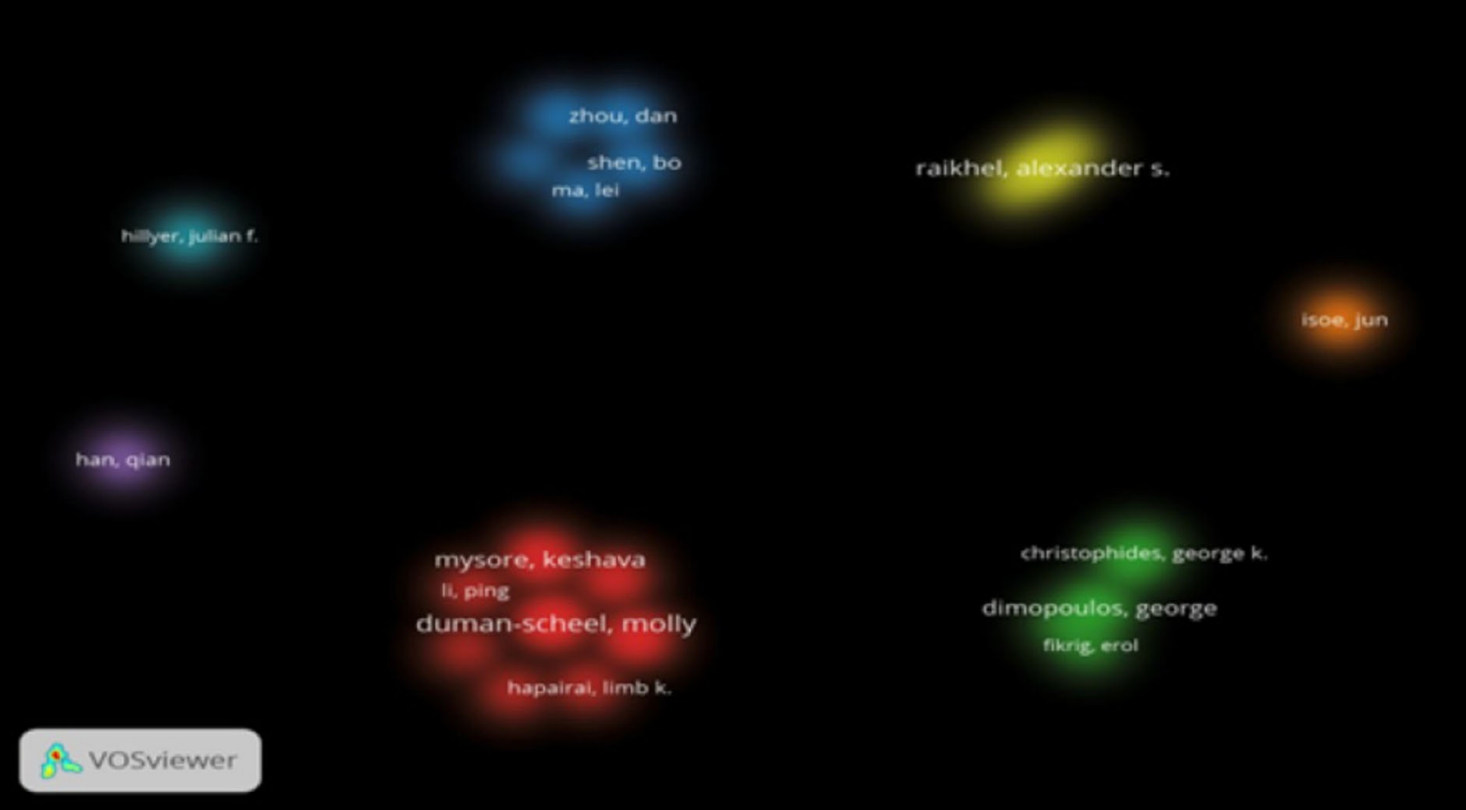
Density visualization map showing co-authorship between authors.

**Figure 5:**
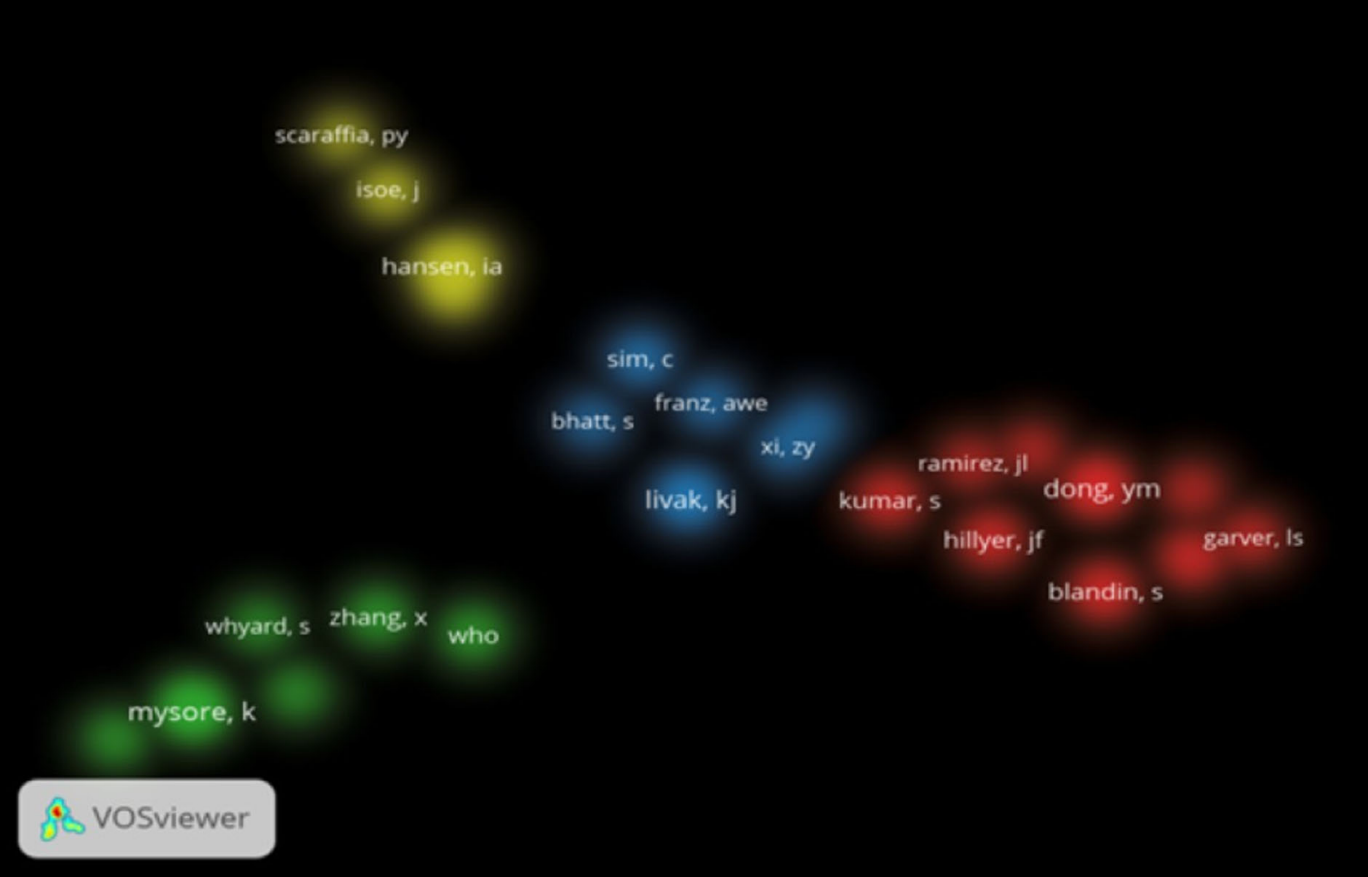
Density visualization map showing co-citation analysis between authors.

The top ten authors were predominantly from the USA, with Duman-Scheel M. (n=27), Mysore K. (n=25), and Severson D.W. (n=23) leading, all affiliated with the University of Notre Dame or Indiana University School of Medicine. China’s Shen B. (n=20) was the only non-US author in the top ten, reflecting China’s emerging role. Co-authorship analysis (VOSviewer, minimum eight documents per author) revealed strong collaborative clusters, particularly among US-based authors, with 24 authors forming distinct research groups (**Figure 4**). Co-citation analysis (minimum 40 citations per author) identified 26 authors, with frequent citations of Duman-Scheel, Mysore, and Raikhel, indicating their significant influence (**Figure 5**). **Table 3** lists the top ten authors in RNAi mosquito research, including their publication counts and institutional affiliations.

**Table 3:** Top 10 leading authors of publications on RNAi in mosquitoes (2010– October 2025).

Rank	Author	Frequency	Affiliation	Country
1^st^	Duman-Scheel M.	27	University of Notre Dame/Indiana University School of Medicine	US
2^nd^	Mysore K.	22	University of Notre Dame/Indiana University School of Medicine	US
3^rd^	Severson DW.	21	University of Notre Dame	US
4^th^	Raikhel AS.	18	University of California, Riverside	US
5^th^	Zhou D.	16	Johns Hopkins University	US
6^th^	Dimopoulos G.	15	Johns Hopkins University	US
7^th^	Shen B.	15	Nanjing Medical University	China
8^th^	Sun L.	15	University of Notre Dame/Indiana University School of Medicine	US
9^th^	Sun Y.	14	Kankas State University	US
10^th^	Isoe J.	12	University of Arizona	US

### 3.5 Analysis of publications based on journals

**Figure 6:**
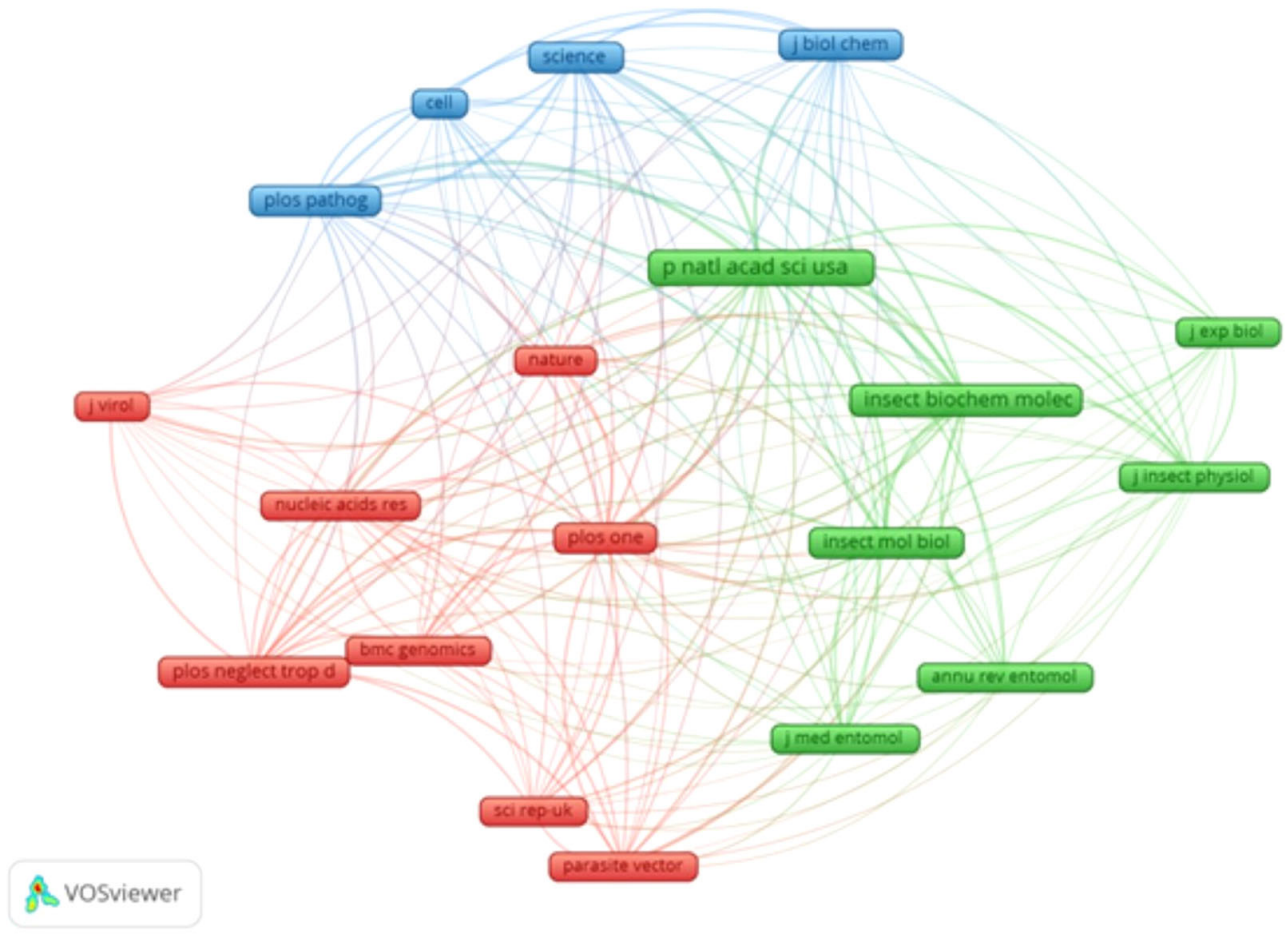
Map of network visualization for co-citations for top journals in publishing articles on RNAi in mosquitoes.

Of 112 journals publishing RNAi mosquito research, *Parasites & Vectors* led with 38 articles, followed by *Insect Biochemistry and Molecular Biology* (n=36) and *PLOS One* (n=35). However, *Proceedings of the National Academy of Sciences* (*PNAS*) had the highest citation impact (n=1483 citations), followed by *PLOS One* (n=1279) and *PLOS Pathogens* (n=1043), reflecting their influence in high-impact research (**Table 4**). Co-citation analysis (VOSviewer, minimum 250 citations) showed strong links between *PNAS* and *Insect Biochemistry and Molecular Biology* (link strength=3122) and *PNAS* and *PLOS Pathogens* (link strength=2857) (**Figure 6**).

**Table 4:** Top 10 journals with the highest publications on RNAi in mosquitoes.

Rank	Sources	TP	TC	H-Index	Impact factor	Quartile
1^st^	Parasites & Vectors	38	666	111	3.06	1^st^
2^nd^	Insect Biochemistry and Molecular Biology	36	826	127	3.2	1^st^
3^rd^	Plos One	35	1279	435	3.11	1^st^
4^th^	Plos Neglected Tropical Diseases	27	839	172	3.4	1^st^
5^th^	Scientific Reports	24	894	315	3.88	1^st^
6^th^	Proceedings of the National Academy of Sciences of the US	22	1483	869	8.18	1^st^
7^th^	Plos Pathogens	18	1043	246	5.44	1^st^
8^th^	Insects	14	63	62	2.87	1^st^
9^th^	Insect Molecular Biology	13	592	102	2.07	1^st^
10^th^	Journal Of Insect Physiology	10	381	115	2.44	1^st^

TP, Total publications; TC, Total citations.

### 3.6 Analysis of most cited articles

The top ten cited articles highlighted key advancements in RNAi mosquito research. Pan et al. (2012) led with 380 citations for their work on *Wolbachia*-induced immune modulation in *Aedes aegypti*, followed by Zhang et al. (2017) (329 citations) on *Aedes aegypti* immunity and Zhang et al. (2010) (325 citations) on nanoparticle-mediated RNAi in *Anopheles gambiae*. Five articles focused on *Aedes aegypti*, four on *Anopheles gambiae*, and one on *Culex quinquefasciatus*, with themes including immune regulation, larval development disruption, and novel delivery systems like chitosan nanoparticles and yeast-based larvicides (**Table 5**).

**Table 5:** Top 10 cited articles on RNAi in mosquitoes (2010– October 2025).

Rank	Title	Author	Year	Source	TC
1^st^	Wolbachia induces reactive oxygen species (ROS)-dependent activation of the Toll pathway to control dengue virus in the mosquito *Aedes aegypti*	PAN X et al	2012	*Proceedings of the National Academy of Sciences*	380
2^nd^	Relish2 mediates bursicon homodimer-induced prophylactic immunity in the mosquito *Aedes aegypti*	ZHANG H et al	2017	*Scientific Reports*	329
3^rd^	Chitosan/double-stranded RNA nanoparticle-mediated RNA interference to silence chitin synthase genes through larval feeding in the African malaria mosquito (*Anopheles gambiae*)	ZHANG X et al (25)	2010	*Insect Molecular Biology*	325
4^th^	Blood Meal-Derived Heme Decreases ROS Levels in the Midgut of *Aedes aegypti* and Allows Proliferation of Intestinal Microbiota	Oliveira Jhm et al	2011	*PLOS Pathogens*	202
5^th^	The *Anopheles gambiae* Odorant Binding Protein 1 (AgamOBP1) Mediates Indole Recognition in the Antennae of Female Mosquitoes	Biessmann H et al	2010	*PLOS One*	181
6^th^	Dengue Virus Infection of the *Aedes aegypti* Salivary Gland and Chemosensory Apparatus Induces Genes that Modulate Infection and Blood-Feeding Behavior	Sim S et al	2012	*PLOS Pathogens*	169
7^th^	Knockdown of a Mosquito Odorant-binding Protein Involved in the Sensitive Detection of Oviposition Attractants	Pelletier J et al	2010	*Journal of Chemical Ecology*	166
8^th^	Chitosan, Carbon Quantum Dot, and Silica Nanoparticle Mediated dsRNA Delivery for Gene Silencing in *Aedes aegypti*: A Comparative Analysis	Das S et al	2015	*ACS Applied Materials & Interfaces*	149
9^th^	The Mosquito Melanization Response Is Implicated in Defense against the Entomopathogenic Fungus *Beauveria bassiana*	Yassine H et al	2012	*PLOS Pathogens*	145
10^th^	Antiviral immunity of *Anopheles gambiae* is highly compartmentalized, with distinct roles for RNA interference and gut microbiota	Carissimo G et al	2015	*Proceedings of the National Academy of Sciences*	129

### 3.7 Analysis of authors’ keywords

**Figure 7:**
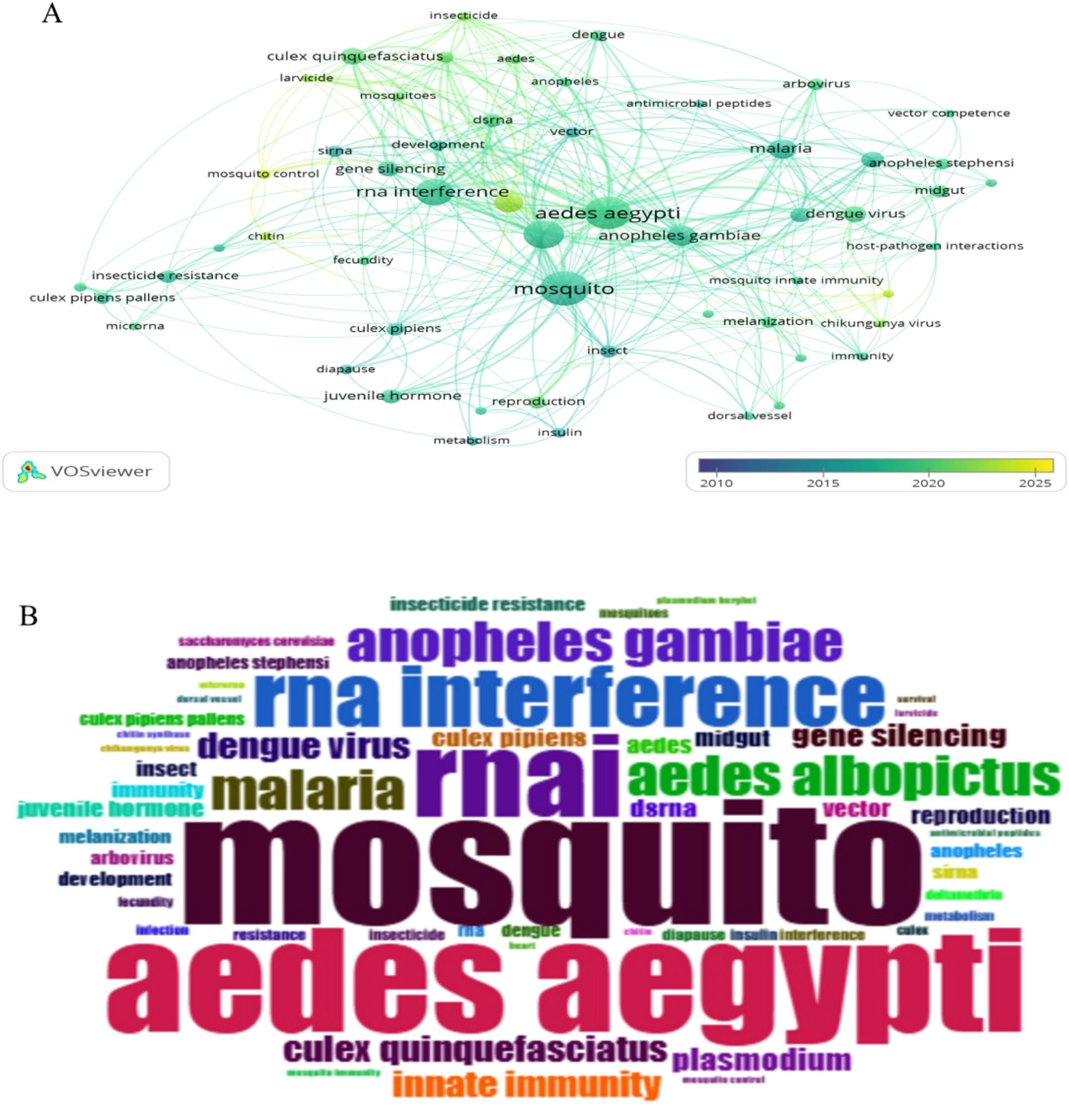
Authors’ keyword analysis **(A)** Overlay visualization of RNAi in mosquito authors’ keyword co-occurrences. **(B)** Most frequently used author keywords.

Keyword co-occurrence analysis (VOSviewer, minimum five occurrences) identified 55 keywords, with “mosquito” (80 occurrences), “*Aedes aegypti*” (70), “RNAi” (53), “RNA interference” (46), and “*Anopheles gambiae*” (26) being the most frequent. An overlay visualization revealed a thematic shift from molecular studies (2010–2014, e.g., gene function, microinjection) to applied technologies (2020–2025, e.g., nanoparticle delivery, RNAi larvicides), reflecting the field’s evolution toward practical vector control (**Figure 7A**). A word cloud further highlighted dominant keywords (**Figure 7B**).

### 3.8 Three-factor analysis

**Figure 8:**
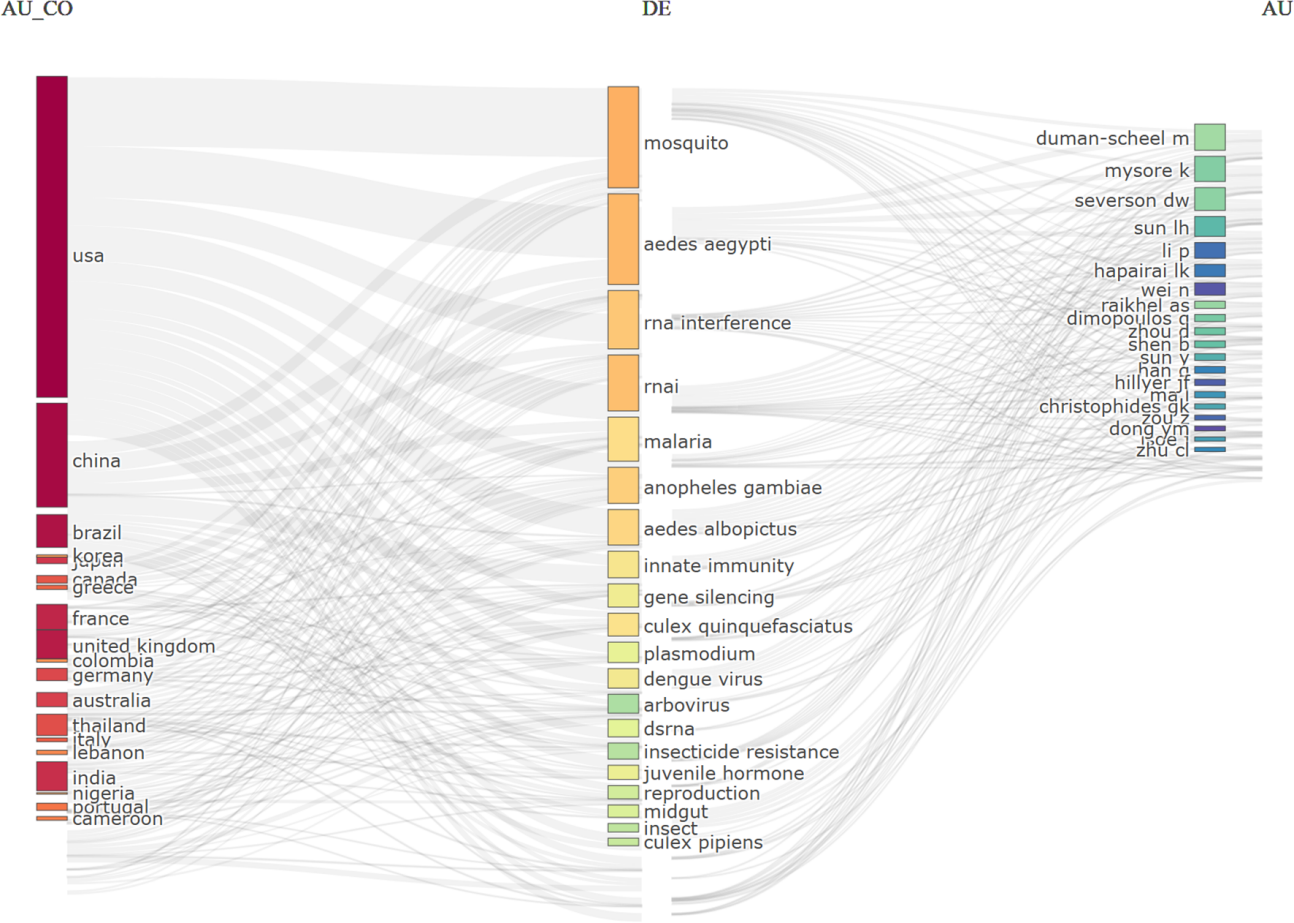
Three-factor analysis of the link between the authors’ Country (left), keywords (center), and authors(right).

A three-factor analysis (Bibliometrix) linked countries, keywords, and authors, revealing that the USA and China dominated keyword usage, particularly “mosquito” and “*Aedes aegypti*” (**Figure 8**). US-based authors like Duman-Scheel and Mysore frequently used these terms, reflecting their research focus.

### 3.9 Implementation of RNAi in vector control

Despite laboratory successes, few RNAi studies have progressed to field validation. **Table 6** summarizes semi-field studies, primarily from the USA and China, testing delivery systems like yeast-based larvicides and attractive toxic sugar baits (18, 28). No African-led field studies were identified, underscoring limited infrastructure and funding (29). Enhanced South-North collaborations are critical to ensure inclusive progress and technology transfer in RNAi-based vector control.

**Table 6:** Field implementation of RNAi studies in mosquito vector control.

Article title	Semi-Field/Field	Country	Author	Reference
A Broad-Based Mosquito Yeast Interfering RNA Pesticide Targeting Rbfox1 Represses Notch Signaling and Kills Both Larvae and Adult Mosquitoes	Semi-field	USA, Trinidad and Tobago	Mysore et al., 2021	(16)
A Yeast RNA-Interference Pesticide Targeting the Irx Gene Functions as a Broad-Based Mosquito Larvicide and Adulticide	Semi-field	USA, Trinidad	Mysore et al., 2021	(13)
Control of Aedes mosquito populations using recombinant microalgae expressing short hairpin RNAs and their effect on plankton	Semi-field	China	Fei et al., 2023	(18)
A Conserved Female-Specific Requirement for the GGT Gene in Mosquito Larvae Facilitates RNAi-Mediated Sex Separation in Multiple Species of Disease Vector Mosquitoes	Semi-field	USA	Mysore et al., 2022	(26)
Generation of a Culex Male Mosquito Sex-Separation RNAi Yeast Strain Using Cas-CLOVER and Super PiggyBac Engineering in Saccharomyces cerevisiae	Semi-field	USA	Brizzee et al., 2023	(27)

### 3.10 Functional categorization of RNAi target genes, RNAi delivery methods and species-specific outcomes

#### 3.10.1 RNAi target genes

Across the analyzed literature, the RNAi target genes are summarized into four major functional categories, including survival, reproduction, development, and metabolic regulation. The studies showed that genes were targeted larvae, pupae and adults as summarized in the **Supplementary Table S1**. Developmental genes such as *chitin synthase 1*, *dopamine 1 receptor (dop1)*, *DOPAL synthase*, and *semaphorin-1a*, among others, were the most frequently targeted (25, 30, 31). For example, RNAi knockdown of *DOPAL synthase* in *Aedes aegypti* larvae caused ~50% mortality and disrupted molting, while *dop1* silencing produced 48–91% higher mortality across *Ae. aegypti*, *Ae. albopictus*, and *An. gambiae* depending on delivery method (microinjection, ATSB, nanoparticle, or yeast shRNA) (30).

Reproductive targets, such as *AaHR78*, *juvenile hormone acid methyltransferase (JHAMT)*, *ammonia transporter*, and *transferrin*, were studied in Aedes species, and their knockdown showed an impact on fertility, fecundity, and ovarian development. Knockdown of *AaHR78* in *Ae. aegypti* led to a significant decrease in egg production (32), while *JHAMT* silencing resulted in 45% inhibition of egg development (33). Also, survival and metabolic regulation genes are presented in the table. Overall, these results showed that RNAi is influenced by different gene functions.

#### 3.10.2 RNAi delivery methods

Delivery methods for RNAi triggers are very crucial for RNAi studies. The reviewed studies showed that microinjection, oral delivery (yeast-expressed shRNA), and nanoparticles are RNAi delivery strategies. Microinjection is the most reliable approach for achieving gene knockdown in mosquitoes, resulting in 48-100% mortality or other phenotypic outcomes. However, it is labor-intensive and unfit for field applications. Nanoparticle-based RNAi, including chitosan or chitosan-TPP formulations, improved RNAi stability and delivery efficiency. For example, chitosan-TPP dsRNA nanoparticles targeting *IAP1* in *Cx. quinquefasciatus* larvae caused 60% mortality (34), while chitosan-dsRNA nanoparticles targeting *chitin synthase 1* in *An. gambiae* increased larval mortality by 26.5% (25). This is a promising approach for field applications. Similarly, oral delivery systems, such as the use of yeast or algae, are promising approaches for field applications.

#### 3.10.3 Species-specific RNAi outcomes

Across the studies, *Ae. aegypti* and *Ae. albopictus*, accounted for the majority of successful experiments, with strong knockdown e.g. *DOPAL synthase*, *dop1*, *semaphorin-1a*, *AaHR78* (30–32, 35). Anopheles species also showed RNAi efficiency in targets such as *arginase*, *trehalase*, and *ecdysone receptor, elongation factor* (15, 36, 37), inducing high mortality and, in other cases, reproductive defects and parasite clearance. Culex species comparatively had fewer studies than any other species, underscoring the need for RNAi investigations in these species.

## 4 Discussion

This bibliometric analysis of RNA interference (RNAi) research in mosquitoes from 2010 to October 2025 provides a comprehensive overview of evolving trends and key developments in this emerging vector control approach. The steady rise in publications, from just 16 in 2010 to peaks of 43 in 2020. This reflects growing global interest in RNAi as an alternative to conventional insecticides (21). Traditional control methods are increasingly limited by resistance and environmental toxicity (8) prompting a shift toward genetic and molecular tools. Notably, the surge in publications aligns with major public health events such as the 2015–2016 Zika virus outbreak, which intensified research on *Aedes* mosquitoes, and with the WHO’s push for sustainable malaria control solutions by 2030 (3, 38). The apparent drop in publications in 2025 (26 articles) likely reflects the dataset’s October cutoff rather than a true decline, underscoring the importance of ongoing monitoring to capture future trends.

The dominance of the United States (45.4% of publications) and China (22.1%) highlights a concentration of expertise and resources in a few countries. Leading institutions such as the University of Notre Dame (n = 98) and Nanjing Medical University (n = 37) have driven much of the global output, supported by strong funding and advanced genomic infrastructure. In contrast, the absence of African countries among the top contributors is striking, particularly given the continent’s disproportionate burden of mosquito-borne diseases. 249 million malaria cases were reported in 2024 alone (3) likely reflects persistent challenges, including limited funding, weak research infrastructure, and reduced visibility of African scholarship in high-impact journals (29). Collaboration patterns reinforce this imbalance: while the USA serves as a central node linking major partners such as China (link strength = 39), African nations remain largely peripheral. Strengthening equitable global partnerships and supporting South–North collaboration could foster technology transfer, local capacity building, and greater inclusion in RNAi innovation—goals consistent with WHO’s call for integrated and inclusive vector control strategies (8).

The prominence of high-impact journals such as *Proceedings of the National Academy of Sciences* (PNAS; 1,483 citations) and *Parasites & Vectors* (38 articles) underscores the field’s scientific visibility. Highly cited studies, including Pan et al. (2012) on *Wolbachia*-induced immunity and Zhang et al. (2010) on nanoparticle-mediated RNAi delivery, reflect a strong focus on immune modulation and delivery technologies. The thematic evolution observed in keyword analyses—from early gene function research (2010–2014) to applied innovations like nanoparticle and yeast-mediated delivery systems (2020–2025)—illustrates the field’s maturation from basic molecular understanding to practical implementation (18, 26). These technological advances, including yeast-based larvicides and attractive toxic sugar baits, highlight RNAi’s potential to disrupt mosquito survival and pathogen transmission with high specificity and minimal ecological risk compared to conventional insecticides (39).

Despite these encouraging trends, several challenges remain. Most RNAi studies are still limited to laboratory settings, with relatively few—mostly from the USA and China—progressing to semi-field validation (e.g (13, 18). Field translation is hindered by persistent technical barriers, such as dsRNA instability and inefficient uptake by wild mosquito populations. Research has also been heavily concentrated on *Aedes aegypti* and *Anopheles gambiae*, leaving important vectors like *Culex quinquefasciatus* and *Mansonia* spp., which transmit diseases such as West Nile virus and lymphatic filariasis, and are understudied. Moreover, potential off-target effects on non-target organisms, including beneficial insects, remain poorly characterized and could present regulatory challenges for RNAi-based biopesticides. Finally, the English-only inclusion criterion may introduce bias, potentially overlooking valuable research from non-English-speaking regions.

RNAi efficacy in mosquitoes depends strongly on the targeted species. Across the analyzed data, *Aedes* species consistently showed RNAi efficacy (30, 34). The successes have been attributed to more cellular uptake and lower nuclease activity. *Anopheles* mosquitoes, on the other hand, showed reduced efficacy to orally delivered dsRNA due to rapid degradation, limited systemic circulation as well as differences in core RNAi machinery components (40). Also, due to limited Culex species studies, generalization is limited, showing a significant knowledge gap in RNAi for these vectors. Uptake in all 3 species was found to be highly dependent on the delivery method. Studies showed that microinjection was reliable in all species as compared to oral and topical delivery in which the dsRNA/siRNA failed to reach internal tissues (40).

The timing of this study is due to the recommendation for novel tools for mosquito vector control, following the problem of resistance and toxicity of chemical insecticides, and the strategic plan to eliminate malaria requires knowledge of what we already have. The findings align with global health priorities, emphasizing the need for innovative and sustainable vector control strategies (8). However, the underrepresentation of African research institutions highlights a critical equity gap that must be addressed through targeted investments and collaborative frameworks. Future studies should focus on developing field-ready formulations, such as RNAi-based sugar baits, and expanding investigations to lesser-studied mosquito species. Establishing clear regulatory pathways will also be essential for the safe and effective deployment of RNAi technologies. By working together across countries and building stronger local research capacity, the global community can help turn RNAi research into real-world solutions—making it a powerful tool for controlling mosquito-borne diseases and protecting public health.

### 4.1 Limitations of RNAi-based mosquito control

Despite RNAi posing as a high efficacious tool for *Aedes* mosquito control, recent studies have raised concerns about its reproducibility and generalizability. Romoli et al. (2024) reported failure of virus block in Aedes species when fed with both naked dsRNA and through bacterial delivery (41). The study attributed this to the lack of the Loqs2 protein in the midgut, which prevents the RNAi machinery from effectively blocking viral replication at the point of entry. Additionally, the mosquito gut and hemolymph contain potent RNases that rapidly degrade double-stranded RNA (dsRNA) before it can be processed into active small interfering RNAs (siRNAs). However, microinjection remains highly effective. Also, Figueiredo Prates et al. (2024) reported inconsistent knockdown effects and failed to reproduce the results reported in articles showing high RNAi efficacy across different delivery methods (42).

These findings suggest that the success of RNAi cannot be assumed across species and even delivery methods, and that previously published literature could be via specific laboratory optimization.

## 5 Conclusion

This bibliometric review highlights significant growth in RNAi research on mosquitoes over the past 15 years. The field has transitioned from fundamental gene-silencing mechanisms to translational applications involving RNAi-based insecticides and nanoparticle delivery systems. While the USA and China remain the global leaders, there is an urgent need to strengthen participation from malaria-endemic regions, particularly in Africa. Future research should focus on developing cost-effective delivery methods, validating field efficacy, and establishing policy frameworks for regulatory approval. Integrating RNAi into national vector control programs could complement existing interventions and help achieve sustainable mosquito management in line with the WHO’s Global Vector Control Response.
