# Supplementary material for: Bibliometric trends and emerging frontiers in RNA interference research for mosquito control (2010–2025)
Source: Front Insect Sci. 2026 Mar 17;6:1758530. doi: 10.3389/finsc.2026.1758530 (PMC13036116; doi:10.3389/finsc.2026.1758530)
Supplement: Supplementary file 1 [file Table1.docx]

**Supplementary Table S1: Summary of Some Key RNAi Studies Included in the Bibliometric Analysis**

| **Authors** | **Year** | **Journal** | **Mosquito species** | **Target gene** | **Functional category** | **Delivery method** | **Reported outcome** | **Ref** |
| --- | --- | --- | --- | --- | --- | --- | --- | --- |
| Mysore et al. | 2021 | Pathogens | *An. gambiae*  *Ae. aegypti*  *Ae. albopictus Cx. quinquefasciatus* | Ataxin 2-binding protein (A2BP1) genes/rbfox1 | Neural Development | Yeast Attractive targeted sugar baits feeding | 93% mortality in *An gambiae,* 77% mortality in *Aedes aegypt*i, 89% mortality in *Aedes albopictus*, 81% mortality in *Cx. quinquefasciatus* | (39) |
| Isoe et al. | 2011 | PNAS | *Ae. aegypti* | Coatomer protein 1 | Intracellular Transport | Microinjection | 88–95% mortality | (40) |
| Mysore et al. | 2020 | PLoS Neglected Tropical Diseases | *Ae. albopictus, Ae. aegypti, An. gambiae, Cx. quinquefasciatus* | *Shaker* gene | Neural/ion channel function | Microinjection & yeast/shRNA attractive sugar bait delivery | 60% mortality in *Ae. albopictus,* 63–64% mortality in *Ae. aegypti,* 53–54% mortality in *An. gambiae,* 58–59% mortality in *Cx. quinquefasciatus* | (41) |
| Deng et al. | 2024 | BMC Genomics | *Ae. albopictus* | PGANT3 | Mucin-type O-glycan biosynthesis / protein glycosylation | dsRNA Microinjection | Reduced Ae. albopictus blood-feeding behavior | (42) |
| Li et al. | 2025 | International Journal of Molecular Sciences | *An. sinensis* | AsCPF1 | Cuticle structure / thickening | dsRNA microinjection | 31.7% increased mortality to deltamethrin | (43) |
| Yan et al. | 2024 | Pest Management Science | Ae. albopictus | AalbOr10 (odorant receptor 10) | Olfactory perception / odorant reception | dsRNA microinjection in adults | Reduced the oviposition repellency behavior | (44) |
| Huang et al | 2025 | Journal of Insect Physiology | Ae. aegypti | AaHR78 (Hormone receptor 78) | Nuclear receptor / endocrine regulation | dsRNA microinjection in larvae & adults) | impaired ovarian development, leading to significantly decreased egg production | (33) |
| Hapairai et al. | 2020 | Insect Biochemistry and Molecular Biology | *Ae. aegypti,*  *Ae. albopictus, An. gambiae* | Dopamine 1 receptor | Neural signaling / neurotransmitter reception | siRNA microinjection, attractive toxic sugar bait (ATSB) delivery (adult), yeast‑expressed shRNA (larval) | 88% mortality in *Ae. aegypti*, ~ 91% mortality in *Ae. albopictus*, ~ 91% mortality in *An. gambiae* for ATSB, ~ 48–49% higher mortality in Ae. aegypti, ~ 55% higher mortality in *Ae. albopictus*, ~ 42% higher mortality rate in *An. gambiae* | (31) |
| Ferdous et al. | 2021 | PLOS Pathogen | *An. coluzzii* | Stearoyl co-A desaturase | Lipid metabolism | dsRNA Microinjection | 100% and 52% mortality after blood feeding with membrane feeder and direct human blood, respectively | (45) |
| Letinić et al. | 2020 | PLoS ONE | *An. arabiensis* | Akirin | Transcription regulation / innate immunity & fitness | siRNA Microinjection | 17% reduction in fecundity rate, 23% reduction in fertility rate, and 23% survival rate | (46) |
| Van Ekert et al. | 2014 | Journal of Insect Physiology | *Aedes aegypti* | Juvenile acid methyl transferase (JHAMT) | Juvenile hormone biosynthesis | dsRNA injection, yeast expressing long hairpin RNA | 45% Egg development inhibition | (34) |
| Maharaj et al. | 2022 | Malaria journal | *An. funestus* | Ecdysone receptor | 20-hydroxyecdysone hormone signaling / nuclear hormone receptor | Microinjection | Only 32% of dsEcR females developed mature eggs | (37) |
| Adedejiet al | 2024 | PLoS ONE | *An. gambiae* | Arginase | Arginine metabolism | Microinjection | Reduced *Plasmodium berghei* oocyst counts in midguts | (15) |
| Durant & Donini | 2020 | PNAS | *Ae. aegypti* | Ammonia transporter/methyl ammonium permease | Ammonia transport / sperm viability & male fertility | Microinjection | 40% reduction in sperm stored in seminal vesicles, resulting in significant decreases in eggs laid per female | (47) |
| Rani et al. | 2022 | PLoS ONE | *An. culicifacies* | Transferrin | Iron transport / reproductive physiology | Microinjection | 23% reduction in oocyst number | (48) |
| Tevatiya et al. | 2020 | Frontiers in Physiology | *An. stephensi* | Trehalase | Carbohydrate metabolism | dsRNA Microinjection | ~50% reduction in eggs laid | (38) |
| Adedeji et al | 2024 | PLoS ONE | *An. gambiae* | elongation factor 2 | Translation elongation | dsRNA Microinjection | Median survival reduced | (15) |
| Adedeji et al | 2024 | PLoS ONE | *An. gambiae* | elongation factor 1 | Translation elongation | dsRNA Microinjection | No significant effect on survival | (15) |
| Chen et al. | 2019 | Parasites & Vectors | *Ae. aegypti* | 3,4-Dihydroxyphenylacetalde hyde synthetase (DOPAL) | Cuticle formation | Microinjection | 50% mortality | (32) |
| Zhang et al | 2010 | Insect Molecular Biology | *An. gambiae* | Chitin synthase 1 | Development | Nanoparticle (chitosan) | Mortality increased by 26.5% in larvae | (30) |
| Dhandapani et al. | 2019 | Scientific reports | *Cx. quinquefasciatus* | Inhibitor of apoptosis 1 (IAP1) | Cell survival | Nanoparticle (chitosan-sodium tripolyphosphate) | 60% mortality of larvae | (35) |
| Mysore et al. | 2013 | PLoS Neglected Tropical Disease | *Ae. aegypti* | Semaphorin 1 a (sema 1a) | Neural development / sensory system development | Nanoparticle | 44% defects in the antenna lobe | (36) |
| Kumar et al. | 2013 | Malaria World Journal | *An. stephensi* | 3-Hydroxy kynurenine transami  nase (3-HKT) | Tryptophan metabolism | Chlamydomonas reinhardtii (algae) | 50% larva mortality | (49) |
